# Comparing patient-reported outcomes and lifestyle factors before and after the COVID-19 pandemic among Black and Hispanic breast cancer survivors in New Jersey

**DOI:** 10.1007/s11764-024-01575-6

**Published:** 2024-04-01

**Authors:** Carola T. Sánchez-Díaz, Nur Zeinomar, Hari S. Iyer, Marley Perlstein, Brian D. Gonzalez, Chi-Chen Hong, Elisa V. Bandera, Bo Qin

**Affiliations:** 1 Section of Cancer Epidemiology and Health Outcomes, Rutgers Cancer Institute of New Jersey, 120 Albany St, New Brunswick, NJ 08901, USA; 2 Rutgers Robert Wood Johnson Medical School, New Brunswick, NJ, USA; 3 Department of Health Outcomes and Behavior, Moffit Cancer Center, Tampa, FL, USA; 4 Department of Cancer Prevention and Control, Roswell Park Comprehensive Cancer Center, Buffalo, NY, USA

**Keywords:** Breast cancer survivors, Hispanic women, Black women, COVID-19, Health-related quality of life, Sleep

## Abstract

**Purpose:**

The impact of the COVID-19 pandemic restrictions in the US since March 2020 on cancer survivorship among Black and Hispanic breast cancer (BC) survivors remains largely unknown. We aimed to evaluate associations of the pandemic with participant characteristics, patient-reported outcomes (PROs), and lifestyle factors among Black and Hispanic BC survivors in the Women’s Circle of Health Follow-Up Study and the New Jersey BC Survivors Study.

**Methods:**

We included 447 Black (*n*_pre_ = 364 and *n*_post_ = 83) and 182 Hispanic (*n*_pre_ = 102 and *n*_post_ = 80) BC survivors who completed a home interview approximately 24 months post-diagnosis between 2017 and 2023. The onset of the pandemic was defined as March 2020. The association of the pandemic with binary outcomes was estimated using robust Poisson regression models.

**Results:**

Hispanic and Black BC survivors recruited after the onset of the pandemic reported higher socioeconomic status and fewer comorbidities. Black women in the post-pandemic group reported a higher prevalence of clinically significant sleep disturbance (prevalence ratio (PR) 1.43, 95% CI 1.23, 1.68), lower sleep efficiency, and lower functional well-being, compared to the pre-pandemic group. Hispanic women were less likely to report low health-related quality of life (vs. high; PR 0.62, 95% CI 0.45, 0.85) after the onset of the pandemic.

**Conclusions:**

Ongoing research is crucial to untangle the impact of the pandemic on racial and ethnic minorities participating in cancer survivorship research, as well as PROs and lifestyle factors.

**Implications for Cancer Survivors:**

This study highlights the importance of considering the impact of the pandemic in all aspects of research, including the interpretation of findings.

## Background

There are limited data on how the COVID-19 pandemic impacted patient-reported outcomes (PROs) and lifestyle factors in breast cancer (BC) survivors of different racial and ethnic groups, particularly after the vaccine became available. Racial and ethnic minoritized populations with cancer are at higher risk of COVID-19 infection, hospitalizations, and more adverse outcomes, compared to their White counterparts [1]. Black and Hispanic BC survivors in the United States (US) experience higher prevalence of obesity [2, 3] and comorbidities [3–5], which, along with the burden of side effects from BC treatment, have been associated with poorer health-related quality of life (HRQoL) [6], sleep disturbance [7], and higher levels of stress [6–9]. Moreover, these comorbidities and lifestyle factors have also been identified as important risk factors for increased COVID-19 burden [4, 10] and compound the physical and psychosocial challenges accompanying BC diagnosis and treatment.

Since the US adopted nationwide pandemic restrictions in March 2020, BC survivors have encountered numerous challenges, including treatment delays, limited access to supportive care, changes in therapeutic encounters, and financial constraints [1, 11–14]. Cancer care disruption may be more pronounced in Black and Hispanic populations, who tend to have lower income occupations and less paid leave for illness given systemic inequities in education and opportunities [12, 15, 16]. Moreover, the pandemic-imposed social distancing restrictions impeded access to vital support networks in these communities. However, taking into account the pronounced cultural importance placed on family and friends within Hispanic and Black women, a favorable trajectory in increased family support during the pandemic could act as an adaptive coping mechanism, enabling these communities to thrive amidst the challenges posed by the ongoing pandemic [17–19].

Given racial health inequities exacerbated during the pandemic, identifying differences in participant characteristics and factors associated with resilience in Hispanic and Black BC survivors could inform post-pandemic studies and strategies to progress toward narrowing disparities in BC survivorship. However, existing studies evaluating the toll of the COVID-19 pandemic on BC survivors are predominantly in White population [20, 21]. Therefore, this study aimed to compare participant characteristics, PROs (i.e., HRQoL, perceived stress, sleep, and financial toxicity), and lifestyle factors (i.e., obesity, smoking, and alcohol) among Black and Hispanic BC survivors interviewed before and after the onset of the COVID-19 pandemic.

## Methods

### Study population and design

We used data from the Women’s Circle of Health Follow-Up Study (WCHFS) [22] and the New Jersey BC Survivors Study (NJBCS) [9, 23], two population-based prospective studies of Black and Hispanic BC survivors in New Jersey. In brief, WCHFS is a longitudinal cohort study of Black BC survivors identified by the New Jersey State Cancer Registry using rapid case ascertainment at 10 counties within the state. Women were eligible to participate if they self-identified as Black or African American, were aged 20–75 years old, had a recent histologically confirmed ductal carcinoma in situ or invasive BC, had no previous history of cancer other than non-melanoma skin cancer, and are able to speak and read English. Baseline and follow-up visits were conducted approximately 10 and 24 months after diagnosis via home interviews, respectively. Home visits included collection of anthropometric measures; blood measures; biospecimen collection, following standardized protocols [22]; and computer-assisted interviews to administer questionnaires assessing sociodemographic, reproductive, and lifestyle factors; PROs; and medical history. Further details regarding the design and conduct of WCHFS have been described previously [22]. For lifestyle factors and PROs in the current analysis for WCHFS participants, only data collected at follow-up visit was used.

The NJBCS, initiated in May 2019, followed a generally similar methodology to that used in the WCHFS [9, 23]. Differences from the WCHFS include six target counties in NJ instead of 10 and only one home visit for data collection approximately 12–24 months after diagnosis, mainly due to budget constraints. Hispanic and Black BC survivors proficient in English and/or Spanish were able to participate in the NJBCS. For Spanish-speaking women, validated Spanish versions of questionnaires were used when possible, and other instruments were professionally translated to Spanish. Data collection followed similar protocols to the WCHFS. First, we confirmed eligibility; then, at the home visit, we collected questionnaires using computer-assisted interviews and body measurements. Both study protocols were approved by the Institutional Review Board at Rutgers, The State University of New Jersey. All participants provided written informed consent before participating.

We included a total of 447 Black and 182 Hispanic BC survivors who completed a home interview ~ 24 months post-diagnosis, from January 2017 to April 2023, 3 years before and after the start of the COVID-19 pandemic restrictions in March 2020. The mean time and SD from diagnosis to interview in the pre- and post-pandemic groups were as follows: pre = 21 months (4.0) and post = 23 months (5.9), for Hispanics, and pre = 26 (3.7) months and post = 23 months (5.9), for Black BC survivors at the follow-up visit in our study. Recruitment of Hispanic and Black BC survivors in the NJBCS was briefly halted due to the unique challenges posed by pandemic restrictions and resumed in December 2020, the same month when the COVID-19 vaccine became available in New Jersey.

### Pandemic period

The COVID-19 pandemic era was defined as “pre-” for participants interviewed during the 3 years preceding the start of the pandemic restriction in March 2020 (January 2017–March 2020), and “post-” for those interviewed within the 3 years after the start of the pandemic restrictions (March 2020–April 2023).

### Patient-reported outcomes

HRQoL was assessed using the Functional Assessment of Cancer Therapy-Breast Cancer (FACT-B) instrument, including five subscale scores physical well-being (PWB), social and family well-being (SFWB), emotional well-being (EWB), functional well-being (FWB), and breast cancer–specific scale (BCS), which is available in English and Spanish [24–26]. Increased FACT-B and domains scores indicate better HRQoL outcome.

Sleep disturbance was assessed using the Pittsburg Sleep Quality Index (PSQI) [27], a 19-item scale that assesses seven components of sleep quality over the past month, including sleep quality, latency, duration, efficiency, habitual sleep disturbance, use of sleep medication, and daytime dysfunction. A score of ≥ 5 for global sleep disturbance and < 85% for sleep efficiency indicates clinically significant outcomes. Sleep efficiency scores were calculated as the percentage of the time spent in bed that is spent asleep [28, 29].

Perceived stress was assessed using the Cohen Perceived Stress Scale 10 (PSS-10) [30]. The PSS asks questions about the participant’s feelings and thoughts during the last month. Finally, to measure financial burden, respondents were asked “To what degree has cancer caused financial problems for you and your family?” with response options ranging from 1 = a lot to 4 = not at all. For all PRO scores in this sample, the internal reliability was generally adequate at each assessment (pre- and post-pandemic), for both Hispanic and Black women (Cronbach’s alpha > 0.70 for all) (Supplementary Table 1).

### Lifestyle factors

Body mass index (BMI) was calculated using weight and height measurements by trained interviewers using a standardized protocol [22, 31], or from self-report if anthropometric measure was not available, defined as weight in kilograms divided by height in meters squared (kg/m^2^). We previously observed high concordance between BMI derived from self-reported weight and height and measured BMI (intraclass correlation = 0.97) [32]. For cigarette smoking, ever smokers were defined as those who reported ever smoking at least one cigarette per day for 1 year. Alcohol consumption since diagnosis was defined based on regular consumption in the year prior to the interview. For drinkers, participants reported information on the type of alcoholic drink (beer, wine, or liquor), the amount, and the frequency consumed. Non-drinkers were defined as those who did not report drinking or drank zero alcoholic beverages per week.

### Statistical analysis

We used the chi-squared test for independence to compare the percentages of patients’ sociodemographic characteristics in each pandemic era group. Continuous variables were compared using the independent two-sample *t*-test.

Multivariable robust Poisson regression models were used to estimate prevalence ratios (PR) with 95% confidence intervals (CI) for the association of enrollment before or after pandemic restrictions with PROs and lifestyle factors. FACT-B score and domains were dichotomized as high (vs. low) based on the sample median. For continuous FACT-B and domains, clinically significant change in HRQoL was defined as a score difference of 7–8 for total score and 2–3 for domains [33]. Obesity was categorized as BMI ≥ 30.0 kg/m^2^. Due to power limitations and to a low prevalence of drinkers reported by this population, alcohol was evaluated as drinking vs. not drinking. Similarly, we evaluated smoking as ever vs. never smoking.

For all models, the covariates of interest considered were age at diagnosis (continuous), educational level (high school or below, some college or higher), annual household income (< $25,000, ≥ $25,000), health insurance (private, Medicare/Medicaid, uninsured/other), marital status (married/living as married, widow/divorced/separated/single), nativity (US-born, foreign-born), menopausal status (premenopausal, post-menopausal), comorbidities (i.e., history of hypertension and diabetes). Covariates included in the multivariable models were selected based on stepwise procedure (*P* value < 0.1).

For Black women, we repeated the analysis among non-Hispanic Black women (*n* = 437). *P* value < 0.05 was considered statistically significant. All statistical analyses were performed using Stata, version 17.0.

## Results

The analytical sample of this population-based study included a total of *n*_pre_ = 102 and *n*_post_ = 80 Hispanic and *n*_pre_ = 364 and *n*_post_ = 83 Black BC survivors. The mean (SD) age for Hispanic and Black BC survivors was 53.5 (10.6) years and 56.6 (10.2) years, respectively.

### Sociodemographic and lifestyle factors and PROs by the COVID-19 pandemic era: descriptive characteristics and univariate comparison

The distribution of relevant sociodemographic characteristics and lifestyle factors by the COVID-19 pandemic era is shown in Table 1. When compared to those in the pre-pandemic group, Hispanic women interviewed after the onset of the pandemic were significantly less likely to have been diagnosed with diabetes (pre- vs. post-pandemic: 28% vs. 10%) and to be drinkers (pre- vs. post-pandemic: 43% vs. 19%) (*P* < 0.05, for all) (Table 1). Hispanic women recruited after the onset of the pandemic reported clinically significant higher overall mean FACT-B score, physical and emotional well-being, and BC symptoms, when compared to the pre-pandemic group (Supplementary Fig. 1).

When compared to the pre-pandemic group, Black BC survivors in the post-pandemic group were significantly more likely to have some college education or higher (pre- vs. post-pandemic: 67% vs. 81%), to be foreign-born (pre- vs. post-pandemic: 15% vs. 29%), and less likely to be diagnosed with hypertension (pre- vs. post-pandemic: 77% vs. 48%) (*P* < 0.05, for all) (Table 1). Black women in the post-pandemic group reported clinically significant lower functional well-being, when compared to the pre-pandemic group (Supplementary Fig. 2). No clinically significant differences in mean scores were observed for the overall FACT-B score and other domains.

### Multivariate comparison of lifestyle factors and PROs pre- and post-pandemic

Results from the multivariable Poisson models indicated that both Hispanic and Black women in the post-pandemic group reported a lower prevalence of moderate to high perceived stress (PR_Hispanic_ 0.85, 95% CI 0.77, 0.94 and PR lack 0.91, 95% CI 0.84, 0.99), and a lower prevalence of alcohol consumption (PR_Hispanic_ 0.41, 95% CI 0.26, 0.68 and PR_Black_ 0.71, 95% CI 0.51, 0.98), compared to the pre-pandemic group (Table 2 and 3).

Multivariable adjusted models also indicated that Hispanic BC survivors in the post-pandemic group were less likely to report low HRQoL (FACT-B total score) (vs. high, PR 0.62, 95% CI 0.45, 0.85) compared to the pre-pandemic group. For FACT-B specific domains, Hispanic women were less likely to report low (vs. high) physical and emotional well-being and BC-specific symptoms (PR 0.71, 95% CI 0.53, 0.94; PR 0.70, 95% CI 0.51, 0.95; and PR 0.59 95% CI 0.43, 0.82, respectively) and more likely to report low social well-being (PR1.32, 95% CI 1.09, 1.60), compared to the pre-pandemic group. No other significant differences by pandemic era were observed for other PROs (i.e., functional well-being, sleep disturbance, efficiency and time, and financial toxicity) and lifestyle factors (i.e., obesity and smoking) among Hispanic women (Table 2).

In contrast, Black BC survivors in the post-pandemic group were more likely to report low functional and emotional well-being (PR 1.41, 95% CI 1.18, 1.69 and PR 1.31, 95% CI 1.08, 1.58, respectively) compared to the pre-pandemic group. Black women also reported a 43% and 68% higher prevalence of clinically significant sleep disturbance and poor efficiency (PR 1.43, 95% CI 1.23, 1.68 and PR1.50, 95% CI 1.30, 1.73, respectively), compared to the pre-pandemic group. No other significant differences by pandemic era were observed for other PROs (i.e., FACT-B total score, physical well-being, social well-being, BC-specific scale, and financial toxicity) and lifestyle factors (i.e., obesity and smoking) among Black women (Table 3). Restricting the analysis to non-Hispanic Black participants did not materially change the results.

## Discussion

In this study, we investigated the potential impact of COVID-19 on participant sociodemographic characteristics, PROs, and lifestyle factors among Hispanic and Black BC survivors in New Jersey. Sociodemographic characteristics and the prevalence of PROs and lifestyle factors varied by pandemic era and race and ethnicity. Hispanic women after the onset of the pandemic reported more favorable overall HRQoL, physical and emotional well-being, and BC symptoms, but lower social well-being, compared to women in the pre-pandemic group. Conversely, Black women in the post-pandemic group had lower functional and emotional well-being, clinically significant sleep disturbance, and lower sleep efficiency compared to the pre-pandemic sample. Hispanic and Black survivors in the post-pandemic group reported lower levels of perceived stress and lower prevalence of alcohol consumption, compared to women in the pre-pandemic group. We also observed important differences in the sociodemographic characteristics of women participating before and after the onset of the pandemic. Hispanic women had lower prevalence of diabetes, while Black women were more educated, were foreign-born, and had a lower prevalence of hypertension, compared to women interviewed before the pandemic. This highlights the possible impact of the pandemic on recruitment in BC survivorship studies and underscores the need of caution in interpreting results.

Contrary to our initial hypothesis, our findings revealed higher levels of HRQoL, physical well-being, emotional well-being, and fewer BC-specific symptoms in the Hispanic post-pandemic group compared to the pre-pandemic group. Yet, lower social and family well-being scores were reported. There are some possible explanations to the higher HRQoL reported by Hispanic women recruited after implementation of pandemic restrictions. First, considering the survivors’ prior adaptation to social distancing restrictions and stress due their diagnosis and treatment, it is possible that the pandemic’s additional restrictions might have had a limited impact on their overall HRQoL and emotional well-being. Moreover, Hispanic survivors may have gone through reconceptualization of their HRQoL in relation to their diagnosis, given the experience of such global natural disaster [34, 35]. Longitudinal studies are needed to evaluate these processes. Moreover, *familism* is a core value within many Hispanic families that have been linked to social support, adaptation [17, 36], and enhanced emotional well-being [37, 38]. However, it is also crucial to recognize that under certain circumstances, familism can inadvertently contribute to negative outcomes [8, 39]. Given the pandemic-induced lack of social proximity to family members and friends during the lockdown, except for those within the same household, it is possible that pre-pandemic poor family values [40] or negative shifts in family dynamics [36, 39] during the pandemic likely contributed to the lower reported social and family well-being in the post-pandemic group. Our results may manifest the complex and diverse effect of family on psychosocial and behavioral outcomes among Hispanic BC survivors [41]. Lastly, these observations could also be a result of self-selection of BC survivors with higher socioeconomic status or fewer comorbidities in the study after the pandemic. Nevertheless, despite the generally higher HRQoL and higher physical, emotional, and BC-specific well-being among Hispanic participants, their overall HRQoL and domain-specific scores remain lower, when compared to Black BC survivors in our study and non-Hispanic White women [8, 42]. These disparities likely stem from multifactorial experiences, including language barriers, access to care, challenges in navigating the healthcare system, and lack of access to culturally and linguistically appropriate clinicians [6, 8].

Additionally, although no significant differences by pandemic era were observed, around 80% and 90% of Hispanic BC survivors reported clinically significant sleep disturbance and low sleep efficiency, which is almost twice the prevalence in the general population [43] and among cancer patients (range 36–69%) [44–46]. Our study underscores the critical need for further investigation beyond the pandemic to comprehend and address the needs of Hispanic BC survivors and narrow these disparities in psychosocial factors.

We also observed worse sleep disturbance and lower functional and emotional well-being among Black women in the post-pandemic group compared to those in the pre-pandemic group. Close to 80% of the Black women in the post-pandemic group reported clinically significant sleep disturbance, which is higher than findings from a prior pre-pandemic study using the full cohort of Black BC survivors in the WCHFS (61%) [7] and exceeding those in a pre-pandemic study in predominantly White BC survivors (42%) [47]. Although studies in Black BC survivors post-pandemic are limited, our results are consistent with a population-based study in the general population that observed the greatest prevalence of post-pandemic sleep disturbance in Black women when compared to other racial and ethnic groups [48]. Moreover, in contrast to prior findings from the WCHFS looking at risk factors for sleep disturbance, where an association of education attainment with sleep disturbance was reported [7], in this study, we observed no differences in the association of COVID-19 pandemic with sleep disturbance and efficiency, when stratified by educational attainment, suggesting that the pandemic impact on sleep disturbance outcomes was irrespective of their educational attainment. This result is not surprising given the heightened social injustice and psychosocial burden of the pandemic among underserved populations, particularly among Black women. For instance, a prior study using a large nationally representative sample of US adults demonstrated a significant association between perceived discrimination during the pandemic and poor sleep quality in Black adults, but not in Hispanic and White adults [49]. Quantitative studies examining the impact of perceived discrimination on sleep in cancer survivorship are warranted. Moreover, sleep disturbance is an important functional impairment in cancer survivors [50] and crucial for their HRQoL, aligning with the poor functional and emotional well-being we observed in the post-pandemic group. A recent study using data from the National Health Interview Survey reported an increased trend in low functional limitations in cancer survivors from 1999 to 2018, including sleep disturbance, disproportionately burdening Black and Hispanic cancer survivors [50]. The continuous assessment of sleep and functional status among Black BC survivors in the post-pandemic era is therefore critical to identify underlying factors explaining these associations and ultimately developing targeted interventions for improved long-term survivorship care. For example, culturally targeted mobile health interventions including cognitive behavioral therapies are promising tools for improving sleep quality among Black [51] and Hispanic [52] BC survivors.

This study has important strengths. The majority of quantitative studies comparing PROs and lifestyle factors before and after the pandemic have been performed early in the pandemic [53]. Therefore, this study stands as one of the few studies comparing PROs and cancer-related behaviors before and after the COVID-19 pandemic, using a diverse population-based study of Black and Hispanic BC survivors. Furthermore, to ensure precise measurement of PROs among an important high-risk population, our study utilized scales with validated Spanish translations specifically tailored for Hispanic women.

This study is not without limitations. First, our results are based on cross-sectional comparisons, and therefore, they should be interpreted with caution. The results from this study are based on the population of Black and Hispanic BC survivors in New Jersey. Therefore, the extent to which our findings can be generalized to other geographic areas in the country is uncertain. Nevertheless, within the state of New Jersey, our study sample demonstrates a high level of diversity and represents the broader population of BC patients. Longitudinal follow-up of the Hispanic and Black BC survivors is warranted to reveal PRO and lifestyle changes over time. We were unable to control for the myriad changes in social patterns that followed pandemic restrictions and occurred alongside the restrictions themselves, which may have influenced the population’s desire and willingness to participate in research. We documented differences in sociodemographic characteristics of participants in our study before and after COVID-19. Despite controlling for these factors, residual confounding is possible. Further research is essential for understanding how to increase the participation of racial and ethnic minority cancer survivors in the post-pandemic era and developing strategies to enhance recruitment. The continuously evolving nature of the pandemic, now considered endemic, suggests that ongoing efforts are necessary.

## Conclusion

The COVID-19 pandemic has introduced additional complexities into the lives of BC survivors, and our findings suggest that its potential impact on research participation, PROs, and lifestyle factors may vary among different racial and ethnic groups. Overall HRQoL remained low, and certain PROs, such as sleep disturbance, were common among both Hispanic and Black BC survivors and remained detrimental compared to other cancer survivors and to the general population. These results underscore the significance of more personalized and culturally informed care practices and highlight the need for healthcare providers to enhance and tailor services to effectively address the diverse needs of Black and Hispanic women. For instance, clinicians could consider assessing sleep quality, recognizing it as a prevalent adverse outcome that persisted among both Hispanic and Black BC survivors even after the onset of the pandemic. Moreover, ongoing evaluation of differences in characteristics of individuals participating in research after the pandemic and further along the cancer continuum, including the survivorship period, is crucial for reducing disparities in minoritized and vulnerable populations.

## Supplementary Material

Supplementary files

**Supplementary Information** The online version contains supplementary material available at https://doi.org/10.1007/s11764-024-01575-6.

## Figures and Tables

**Table 1:** Distribution of sociodemographic characteristics and lifestyle factors by the COVID-19 pandemic era among Black and Hispanic breast cancer survivors in the WCHFS and NJBCS

	Hispanic (*n* = 182)						Black (*n* = 447)					
		
		Pre-pandemic		Post-pandemic				Pre-pandemic		Post-pandemic		
						
		*n* = 102		*n* = 80				*n* = 364		*n* = 83		
						
	Total *n*	*n*	%	*n*	%	*P* value	Total *n*	*n*	%	*n*	%	*P* value

Age at diagnosis												
*mean* (*SD*)		53.7 (10.6)		53.3 (10.6)		0.76		56.9 (10.3)		56.3 (10.1)		0.76
< 45	44	23	23	21	26		157	50	14	14	17	
45–60	57	34	33	23	29		151	155	43	34	41	
≥ 60	81	45	44	36	45		139	159	44	35	42	
Education						0.37						0.01
High school grad or below	100	59	58	41	51		134	118	33	16	19	
Some college or higher	82	43	42	39	49		312	245	67	67	81	
Household income (USD)						0.18						0.67
< 25,000	70	51	53	19	41		111	96	30	15	27	
≥ 25,000	72	45	47	27	59		270	229	71	41	73	
Health insurance						0.04						0.94
Private	67	31	30	36	45		238	193	53	45	54	
Medicare/Medicaid	68	38	37	30	38		168	138	38	30	36	
Uninsured/other	47	33	32	14	18		41	33	9	8	10	
Marital status						0.51						0.83
Married/living as married	99	58	57	41	52		167	135	37	32	39	
Not married or living as married	82	44	43	38	48		278	227	63	51	61	
Foreign born						0.27						0.001
No	26	12	12	14	18		368	311	85	57	69	
Yes	156	90	88	66	82		77	53	15	24	29	
Menopausal status						0.75						0.84
Premenopausal	66	38	37	28	35		144	118	32	26	31	
Post-menopausal	116	64	63	52	65		303	246	68	57	69	
Body mass index												
*mean* (*SD*)		29.8 (4.8)		29.4 (6.7)		0.35		31.9 (6.6)		31.0 (6.4)		0.55
Normal/underweight	34	15	15	19	24		67	52	14	15	18	
Overweight	67	41	40	26	32		115	95	26	20	24	
Obese	81	46	45	35	44		264	216	60	48	58	
Diabetes						< 0.001						0.48
No	145	73	72	72	90		337	272	75	65	78	
Yes	37	29	28	8	10		110	92	25	18	22	
Hypertension						0.72						< 0.001
No	118	65	64	53	66		128	85	23	43	52	
Yes	64	37	36	27	34		319	279	77	40	48	
Alcohol since diagnosis						< 0.001						0.09
Non-drinkers	123	58	57	65	81		169	113	54.3	56	65.9	
Any	59	44	43	15	19		122	95	45.7	27	31.8	
Smoking						0.44						0.17
Never	134	76	74.5	58	72.5		280	221	61	59	71	
Ever	42	26	25.5	21	27.5		167	143	39	24	29	

**Table 2:** Associations of the COVID-19 pandemic era (pre- vs. post-) with patient-reported outcomes and lifestyle factors among Hispanic breast cancer survivors in the NJBCS^1^

Patient-reported outcomes^2^	Hispanic (*n* = 182)		
	
COVID-pandemic era	PR	95% CI	

FACT-B total score (low vs. high)			
Pre-pandemic		Ref	
Pandemic	0.62	0.45	0.85
Physical well-being (low vs. high)			
Pre-pandemic		Ref	
Pandemic	0.71	0.53	0.94
Functional well-being (low vs. high)			
Pre-pandemic		Ref	
Pandemic	1.07	0.84	1.38
Social well-being (low vs. high)			
Pre-pandemic		Ref	
Pandemic	1.32	1.09	1.60
Emotional well-being (low vs. high)			
Pre-pandemic		Ref	
Pandemic	0.70	0.51	0.95
Breast cancer subscale (low vs. high)			
Pre-pandemic		Ref	
Pandemic	0.59	0.43	0.82
Perceived stress (moderate/high vs. low)			
Pre-pandemic		Ref	
Pandemic	0.85	0.77	0.94
Clinically significant sleep disturbance (PSQI ≥ 5)			
Pre-pandemic		Ref	
Pandemic	0.89	0.77	1.03
Sleep duration (hours) (< 7 and ≥ 9 vs. 7–8)			
Pre-pandemic		Ref	
Pandemic	1.05	0.81	1.36
Clinically significant sleep efficiency (≤ 85%)			
Pre-pandemic		Ref	
Pandemic	1.02	0.94	1.11
Financial toxicity			
Pre-pandemic		Ref	
Pandemic (some vs. not at all)	0.88	0.68	1.17
Pandemic (a lot vs. not at all)	1.14	0.87	1.49
Lifestyle factors			
Alcohol since diagnosis (drinkers vs. non-drinkers)			
Pre-pandemic		Ref	
Pandemic	0.41	0.26	0.68
Ever smoking (yes vs. no)			
Pre-pandemic		Ref	
Pandemic	1.04	0.65	1.71
Obesity			
Pre-pandemic		Ref	
Pandemic	0.99	0.72	1.37

*FACT-B* Functional Assessment of Cancer Therapy-Breast Cancer, *PSQI* Pittsburgh Sleep Quality Index

1 Robust Poisson regression models were used to evaluate the association of COVID-19 era with categorical patient-reported outcomes and lifestyle factors. All models were adjusted by age and education

2 FACT-B and domains were categorized as high (vs. low) based on sample median

**Table 3:** Associations of the COVID-19 pandemic era (pre- vs. post-) with patient-reported outcomes and lifestyle factors among Black breast cancer survivors in the WCHS and NJBCS^1^

Patient-reported outcomes^2^	Black (*n* = 447)		
	
COVID-pandemic era	PR	95% CI	

FACT-B total score (low vs. high)			
Pre-pandemic		Ref	
Pandemic	1.10	0.88	1.37
Physical well-being (low vs. high)			
Pre-pandemic		Ref	
Pandemic	0.98	0.79	1.22
Functional well-being (low vs. high)			
Pre-pandemic		Ref	
Pandemic	1.41	1.18	1.69
Social well-being (low vs. high)			
Pre-pandemic		Ref	
Pandemic	0.93	0.73	1.17
Emotional well-being (low vs. high)			
Pre-pandemic		Ref	
Pandemic	1.31	1.08	1.58
Breast cancer subscale (low vs. high)			
Pre-pandemic		Ref	
Pandemic	0.84	0.65	1.08
Perceived stress (moderate/high vs. low)			
Pre-pandemic		Ref	
Pandemic	0.91	0.84	0.99
Clinically significant sleep disturbance (≥ 5)			
Pre-pandemic		Ref	
Pandemic	1.43	1.23	1.68
Sleep duration (hours) (< 7 and ≥ 9 vs. 7–8)			
Pre-pandemic		Ref	
Pandemic	0.78	0.64	0.97
Clinically significant sleep efficiency (≤ 85%)			
Pre-pandemic		Ref	
Pandemic	1.50	1.30	1.73
Financial toxicity			
Pre-pandemic		Ref	
Pandemic (some vs. not at all)	0.92	0.63	1.35
Pandemic (a lot vs. not at all)	1.07	0.78	1.47
Lifestyle factors			
Alcohol since diagnosis (drinkers vs. non-drinkers)			
Pre-pandemic		Ref	
Pandemic	0.71	0.51	0.98
Ever smoking (yes vs. no)			
Pre-pandemic		Ref	
Pandemic	0.76	0.53	1.09
Obesity			
Pre-pandemic		Ref	
Pandemic	0.98	0.80	1.20

*FACT-B* Functional Assessment of Cancer Therapy-Breast Cancer, *PSQI* Pittsburgh Sleep Quality Index

1 Robust Poisson regression models were used to evaluate the association of COVID-19 era with categorical patient-reported outcomes and lifestyle factors. All models were adjusted by age and education

2 FACT-B and domains were categorized as high (vs. low) based on sample median

## Data Availability

No datasets were generated or analyzed during the current study.
