# Supplementary material for: Comparing patient-reported outcomes and lifestyle factors before and after the COVID-19 pandemic among Black and Hispanic breast cancer survivors in New Jersey
Source: J Cancer Surviv. 2024 Apr 1;19(5):1588–97. doi: 10.1007/s11764-024-01575-6 (PMC12449782; doi:10.1007/s11764-024-01575-6)
Supplement: Supplementary file 1 — Supplementary file1 (DOCX 26 KB) [file 11764_2024_1575_MOESM1_ESM.docx]

**Supplementary files**

Supplementary Table 1. Internal consistency reliability for pre- and post- patient-reported outcome scales among Hispanic and Black breast cancer survivors.

|  | Cronbach’s alpha | | | |
| --- | --- | --- | --- | --- |
|  | Hispanic | | Black | |
|  | pre- | post- | pre- | post- |
| FACT-B total score | .93 | .89 | .83 | .90 |
| Cohen-Perceived stress | .83 | .84 | .85 | .80 |
| PSQI Score | .77 | .78 | .79 | .67 |

FACT-B: Functional Assessment of Cancer Therapy-Breast Cancer; PSQI: Pittsburgh Sleep Quality Index.

Supplementary Figure 1. Means of FACT-B total score and domains by COVID-19 pandemic era among Hispanic breast cancer survivors in New Jersey.

**Clinically significant mean difference in FACT-B is 7-8 for total score and 2-3 for domains. PWB = physical well-being. FWB = functional well-being. SFWB = social well-being. EWB = emotional well-being. BCS = breast cancer symptoms.

Supplementary Figure 2. Means of FACT-B total score and domains by COVID-19 pandemic era among Black breast cancer survivors in New Jersey.

**Clinically significant mean difference in FACT-B is 7-8 for total score and 2-3 for domains. PWB = physical well-being. FWB = functional well-being. SFWB = social well-being. EWB = emotional well-being. BCS = breast cancer symptoms.
